# The role of orthodontics in the prevention and management of gingival recession

**DOI:** 10.1038/s41415-024-7781-1

**Published:** 2024-09-13

**Authors:** ﻿Padhraig S. Fleming, James Andrews

**Affiliations:** 41415199179001 Chair/Professor of Orthodontics, School of Dental Science, Dublin Dental University Hospital, The University of Dublin, Trinity College Dublin, Ireland; Honorary Professor of Orthodontics, Queen Mary University of London, UK; 41415199179002 Specialist in Orthodontics, Perth, Western Australia, Australia

## Abstract

Careful management of orthodontic patients presenting with thin periodontal phenotype is paramount. Combined orthodontic-periodontal input is helpful both in terms of diagnosis and stabilisation but also to coordinate care. Well-executed orthodontics offers the potential to safeguard periodontal health but also to induce significant aesthetic improvement either in isolation or combined with increasingly predictable muco-gingival procedures.

## Key points

- Thin periodontal phenotype is associated with increased risk of recession during orthodontic treatment necessitating detailed planning and indicating combined orthodontic-periodontal input in selected cases with a clear understanding of the optimal nature and timing of interventions an imperative.
- Careful space creation and judicious and flexible use of mechanics are essential in ensuring optimal tooth positioning in order to ensure optimal long-term outcomes.
- Carefully-planned orthodontics is generally compatible with periodontal health even in susceptible patients. Moreover, orthodontics in isolation or in combination with muco-gingival surgery may contribute to enhanced periodontal outcomes.

## Introduction

Gingival recession involves exposure of the root surfaces due to apical migration of the gingival margin relative to the cemento-enamel junction. The prevalence of recession is age-related with a predilection among adults particularly over the age of 50 years.^1^ Recession is inextricably linked with hard tissue loss with 1 mm of recession associated with 2.8 mm of bone dehiscence.^2^ Each further 1 mm increment has been linked to a commensurate (0.98 mm) amount of dehiscence.^2^ There are a myriad of contributors including periodontal disease and hygiene measures allied to maturational changes including declining vascularity and collagen content in gingival tissues. The increasing traction of adult orthodontics has prompted an onus on the management of both pre-existing recession and susceptible patients within routine clinical practice. Orthodontic treatment can undoubtedly induce unwanted recession particularly in this cohort. There is also an increased risk associated with ambitious tooth movement outside the alveolar boundaries in all patients. In growing patients, this may not be obvious during or even immediately after treatment; however, this approach may represent a significant risk factor for recession in adulthood.^2^ Conversely, carefully planned treatment can be used as a means of preventing deterioration or indeed in addressing recession either independently or in combination with periodontal therapy.

## Diagnosis and classification

Recession is typically diagnosed clinically and may be associated with aesthetic impact as well as sensitivity. Clinical parameters include recession depth, probing pocket depths, clinical attachment level, and width and thickness of keratinised tissue. Supplementary imaging including two-dimensional intra-oral views and cone-beam computed tomography (CBCT) may provide additional information on inter-proximal bone heights, bone volume and topography including the presence of fenestration and dehiscence. An accepted classification of recession based on the gingival margin height relative to the mucogingival junction and accounting for inter-proximal bone and soft tissue loss was proposed by Miller.^3^ This has since been updated to encompass both mid-buccal or mid-lingual attachment levels relative to the inter-proximal bone but also the width of the attached gingiva and gingival thickness with 1 mm thresholds for the latter two parameters.^4^

## Orthodontic planning and recession

Orthodontic treatment is proven to induce predictable aesthetic improvement. This may translate into social and socio-psychological benefit particularly in those with more salient features of malocclusion in the aesthetic zone.^5^^,^^6^^,^^7^ There is, however, associated risk including: root resorption, demineralisation and periodontal issues. The potential aesthetic benefit of treatment should therefore be considered in this context with risk factors for deleterious effects being identified and mitigated.

Gingival recession may emanate from undermined periodontal support with the cortical plates being largely immutable. This can be potentiated by significant anteroposterior and transverse dento-alveolar change during treatment but also during the resolution of crowding, which may induce incisal proclination and transverse expansion.^8^ Significant arch lengthening can lead to resorption of the cortical plates resulting in fenestration and dehiscence with the latter involving the alveolar margin. It is, however, noteworthy that the prevalence of both labial fenestration and dehiscence is high in untreated subjects at 36% and 20%, respectively.^9^ Based on CBCT, fenestrations were more common on the canine teeth and most prevalent in the apical third but involved the entire root in 8.4% with palatal dehiscence detected in less than 2% in the anterior maxilla.^9^

Thresholds for safe orthodontic tooth movements are imposed both by the constraints related to the alveolar housing, cortical plates and investing soft tissues. In addition to periodontal compromise, violation of these limits may risk both root resorption and instability.^10^ It is accepted that a narrow band of as little as 1 mm of keratinised attached gingival tissue may be sufficient to withstand orthodontic stresses.^11^^,^^12^ Equally, by preserving the position of the teeth within the alveolar process, the risk of recession is minimised.^11^^,^^12^

## Periodontal phenotype

**Fig. 1:**
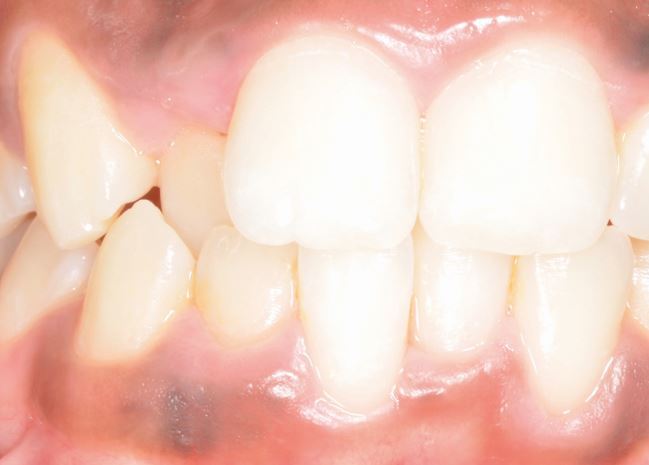
A crowded dentition with palatal displacement of 12 and lingual positioning of 42 and 31. Note the excess gingival tissue present. Conversely, both 41 and 32 are labially placed with associated recession

**Fig. 2:**
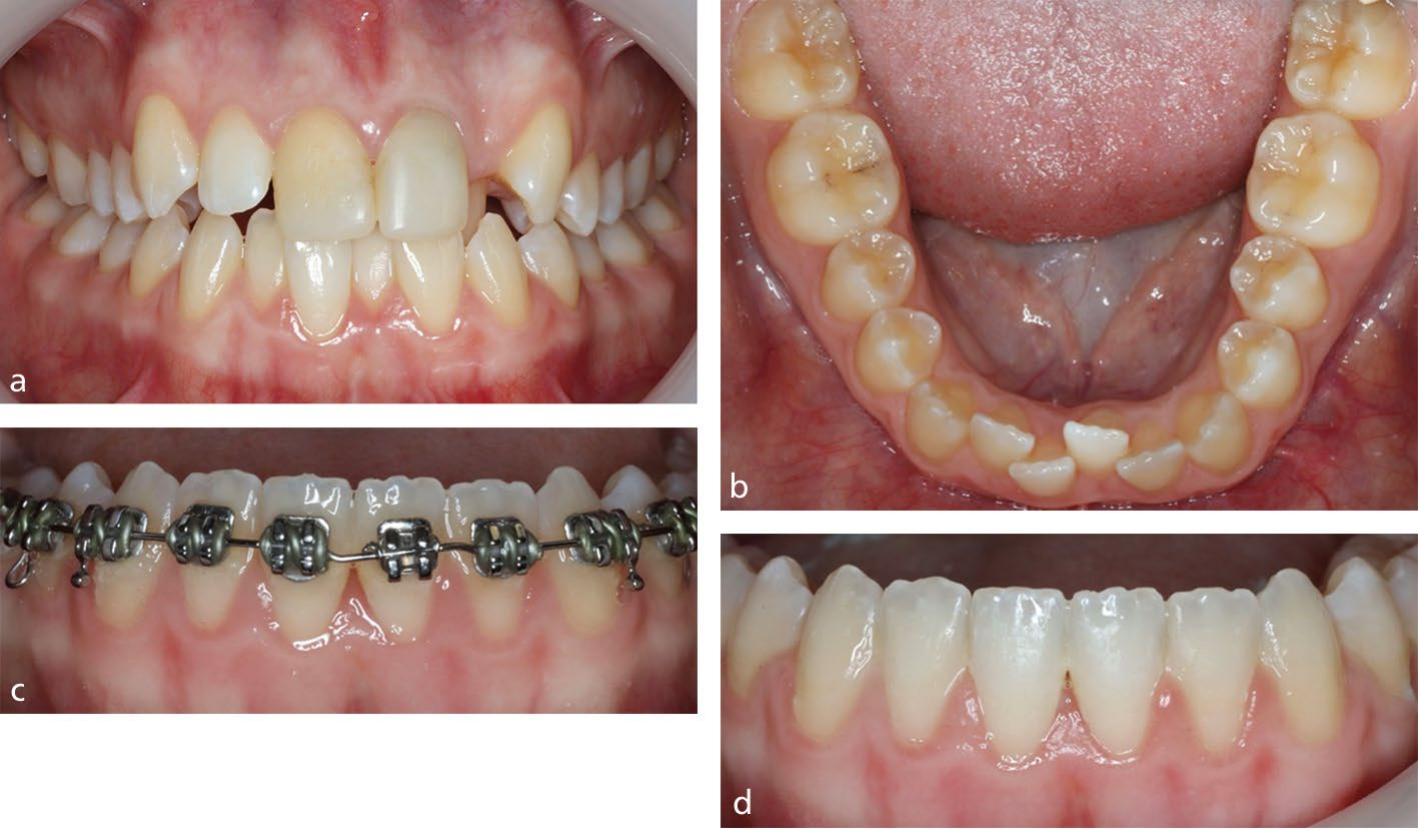
a, b) Both 41 and 32 were labially displaced, reflecting lower anterior malalignment. c, d) Following simple re-alignment involving judicious local space creation, the lower anteriors were aligned with improvement in the gingival appearance reflecting repositioning within the alveolar housing

Recession is particularly likely in those with thin periodontal biotype or phenotype. The term ‘phenotype' has been advanced as biotype reflects genetically predetermined appearance, while phenotype might also encompass environmental influences including orthodontics, mucogingival procedures and overhanging restorations.^13^ A thin phenotype can be diagnosed visually but also during probing with visibility of the periodontal probe expected with a thickness below 1 mm.^14^ Vertical facial dimension may be associated with phenotype although this has not uniformly been demonstrated in younger adults and orthodontic patients.^15^^,^^16^ From a clinical perspective, it is important to appreciate the relationship between tooth position and gingival coverage. Specifically, labially-displaced and rotated teeth may lack gingival coverage labially, while lingually-positioned teeth may present with bunching of tissue labially (Figures 1 and 2).

## Orthodontic diagnosis in the susceptible periodontium

Tailored orthodontic diagnosis has been facilitated by the advent and increasing adoption of CBCT. While blanket prescription of cone-beam imaging remains exceptional governed by dose limitation,^17^ detailed imaging may help to isolate patients or sites that are more susceptible to recession. It is accepted, however, that periodontal ligaments spaces of 200 μm or less may not be detectable risking false positive findings of fenestration or dehiscence. Moreover, image resolution is affected by patient motion, reduced spatial resolution at the periphery and voxel size with a smaller voxel size (<100 μm) leading to enhanced resolution but with an attendant increase in radiation dosage.^17^^,^^18^

**Fig. 3:**
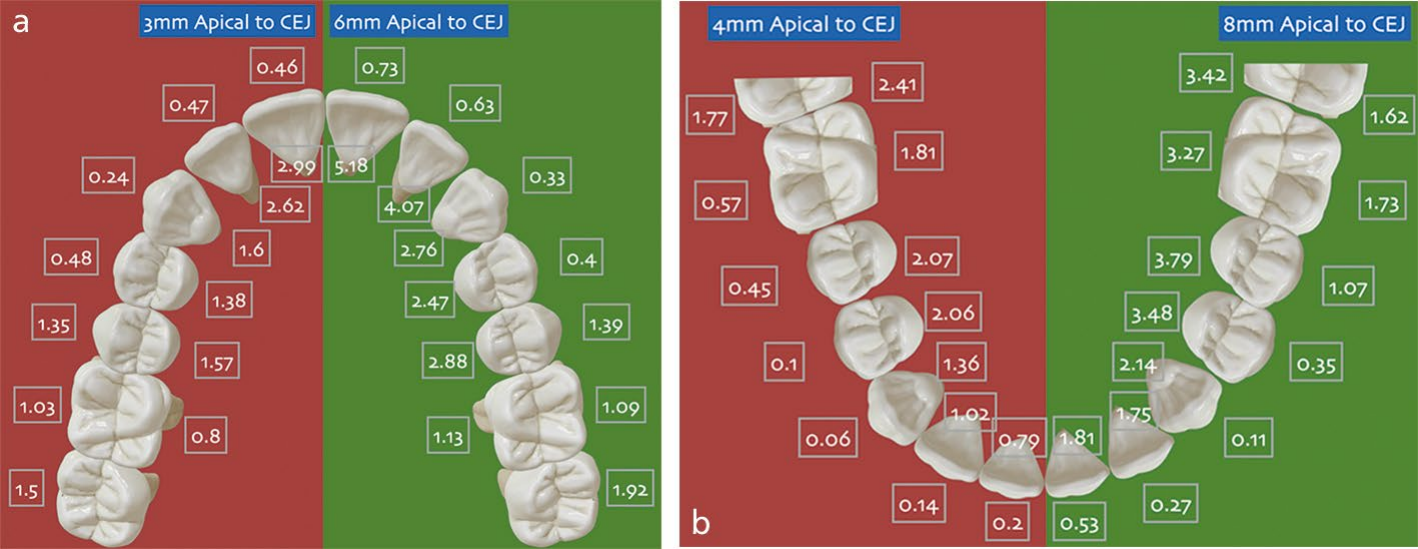
a, b) Alveolar plate thickness (mm) from 3 mm to 8 mm apical to the cemento-enamel junction. Note that the labial plates are thinner than palatal particularly in the inter-first premolar region^19^

From a research perspective, CBCT has been instructive in mapping alveolar boundaries highlighting that cortical plates are generally thin even in adolescence but particularly on the labial aspects of the teeth.^19^ Cortical plates tend to be particularly thin in the labial inter-canine region in both upper and lower arches (Fig. 3). Deleterious change may therefore be risked with significant mandibular incisor proclination, in particular. Based on analysis of 49 subjects using baseline and post-treatment CBCTs, Matsumoto *et al*. (2020)^20^ reported a high prevalence of dehiscence. In an adolescent sample with a mean age of 11.2 years, dehiscence was present at baseline on 32% of mandibular incisors in male patients and 24% of teeth in female patients. These prevalence rates almost doubled (to 58% in male and 45% in female patients) following treatment. Proclination of the mandibular incisors predisposed to dehiscence, with a 50% probability of 2 mm of vertical bone loss following lower incisor proclination of 8 degrees.^20^

While CBCT may be particularly instructive in certain situations, adjunctive use remains best reserved for more susceptible patients and sites. In the absence of three-dimensional volumetric information, the recommended scope of tooth movement can only be approximated. However, significant recession on both labial and lingual aspects in the presence of a thin periodontal phenotype may contra-indicate significant tooth movement without adjunctive periodontal treatment. Conversely, recession confined to a single surface may dictate a more assertive approach.

## Surgical root coverage procedures

Gingival augmentation procedures include flaps and grafts. Significant advancements in the predictability of graft procedures have occurred in recent years with subepithelial connective tissue grafts from intra-oral sites, the use of novel soft tissue substitutes, and periodontal soft tissue phenotype modification gaining increased traction.^21^ Phenotypic modification includes histologic and clinical changes induced by soft tissue grafts characterised by thickening of the epithelial layer, and an increase in the number and density of collagen bundles in the lamina propria. This translates into increased width and thickness of the keratinised tissue enhancing the prospect of complete root coverage and gingival stability with five-year follow-up indicating that a minimum threshold of 2 mm width of attached gingiva and 1 mm thickness is required to maintain marginal stability in both treated and untreated groups.^22^

Evidence supporting the effectiveness of root coverage procedures is clear-cut, with subepithelial connective tissue grafting in conjunction with coronally advanced flaps being indicated both for single and multiple recession sites based on follow-up of up to 12 months.^23^ Factors influencing the scope and remit of subepithelial grafting include the location and number of defects, the thickness and width of the flap, and the volume of graft required from donor sites including the palate and tuberosity.

In instances where the width and thickness of gingival tissue or keratinised tissue rather than root coverage is required, free gingival grafts are preferable with subepithelial grafting using a tunnelling approach also proven effective.^22^ Free gingival grafts are best reserved for sites with lower aesthetic premium including mandibular sites due to changes in texture and lighter colour. Subepithelial grafts may be better suited to more aesthetic regions including the labial aspect of the maxillary incisors.^24^

## Combined orthodontic-periodontal planning

**Fig. 4:**
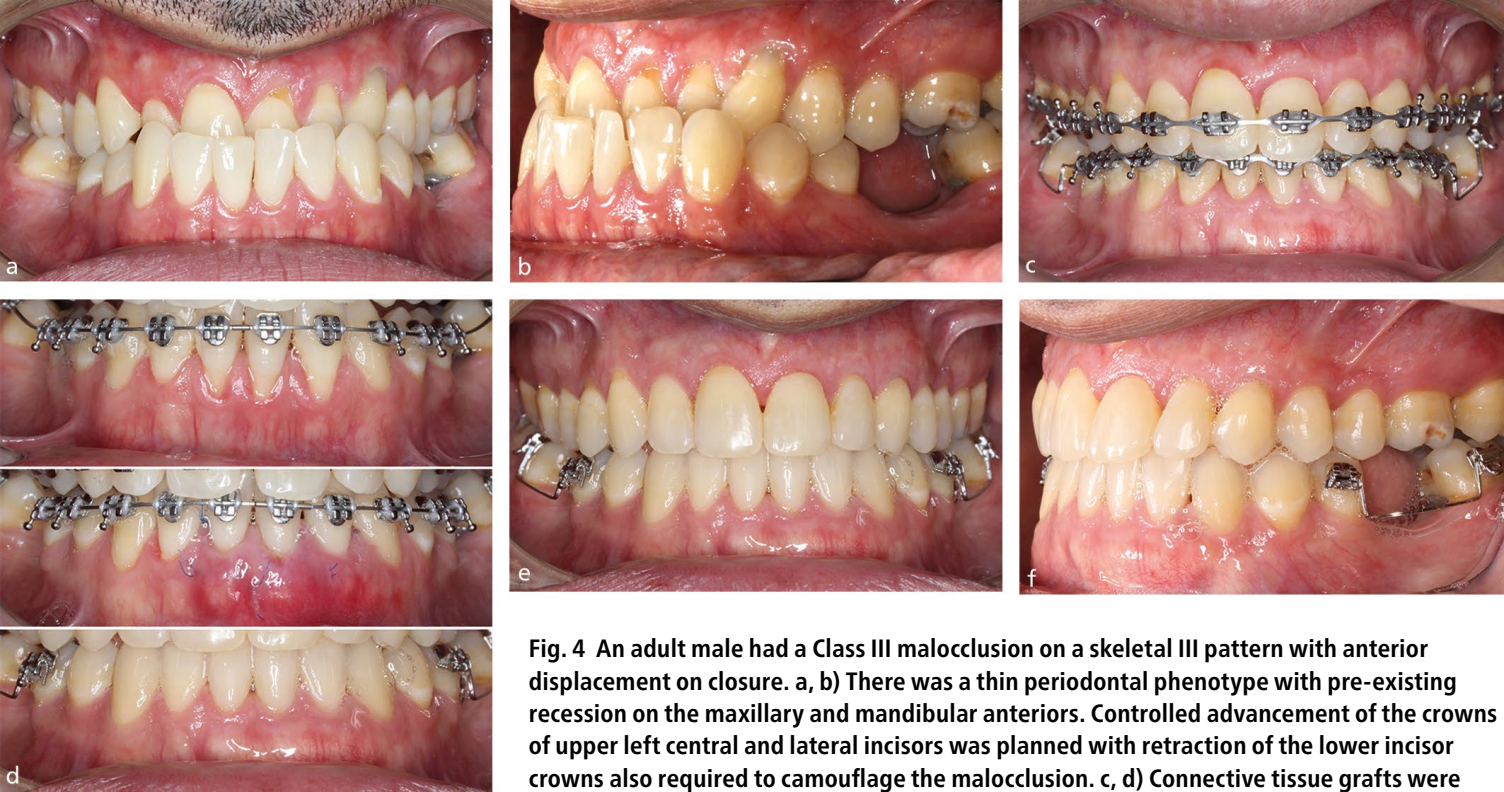
An adult male had a Class III malocclusion on a skeletal III pattern with anterior displacement on closure. a, b) There was a thin periodontal phenotype with pre-existing recession on the maxillary and mandibular anteriors. Controlled advancement of the crowns of upper left central and lateral incisors was planned with retraction of the lower incisor crowns also required to camouflage the malocclusion. c, d) Connective tissue grafts were undertaken in the upper left quadrant and lower incisor region labially during treatment. e, f) Full occlusal correction was achieved with the overjet normalised. Further grafting in the upper right quadrant may be considered in time.

The principles underpinning orthodontic treatment generally apply to those with susceptible periodontal phenotype. In particular, however, the risk of deleterious change associated with injudicious arch lengthening by proclination or expansion is paramount. Moreover, a tooth-specific assessment of the risks of treatment may be warranted based both on the features of the malocclusion, patient concerns, individual susceptibility and local anatomical factors. Inter-disciplinary evaluation may be appropriate in patients with mucogingival deformities with periodontal health before the commencement of treatment a prerequisite. Where gingival augmentation procedures are performed, wound healing should be permitted over a period of at least six weeks and three months following free gingival grafting and sub-epithelial connective tissue grafting, respectively. Thereafter, the impact of buccal or labial orthodontic tooth movement should be considered with regular periodontal maintenance and joint post-treatment evaluation recommended (Fig. 4).^25^

Clinical data underpinning the appropriate staging of periodontal and orthodontic therapy in the presence of muco-gingival defects are lacking. Undoubtedly, roots positioned labial to the alveolar housing should initially be aligned and appropriately torqued before soft tissue augmentation. Conversely, however, in the presence of a particularly thin alveolus, this procedure may risk the creation of a dehiscence on the lingual aspect.^21^ The institution of root coverage procedures before orthodontics has been associated with improved stability of marginal levels and an improved width of keratinised tissue relative to deferral.^26^ It therefore appears reasonable to recommend prior grafting procedures with recession defects characterised by minimal (<1 mm) width of attached gingiva and gingival thickness.^21^ Exceptions to this approach include extra-alveolar root positioning and excessively thin alveolar dimensions.

## Orthodontic planning and mechanics

### Space creation

**Fig. 5:**
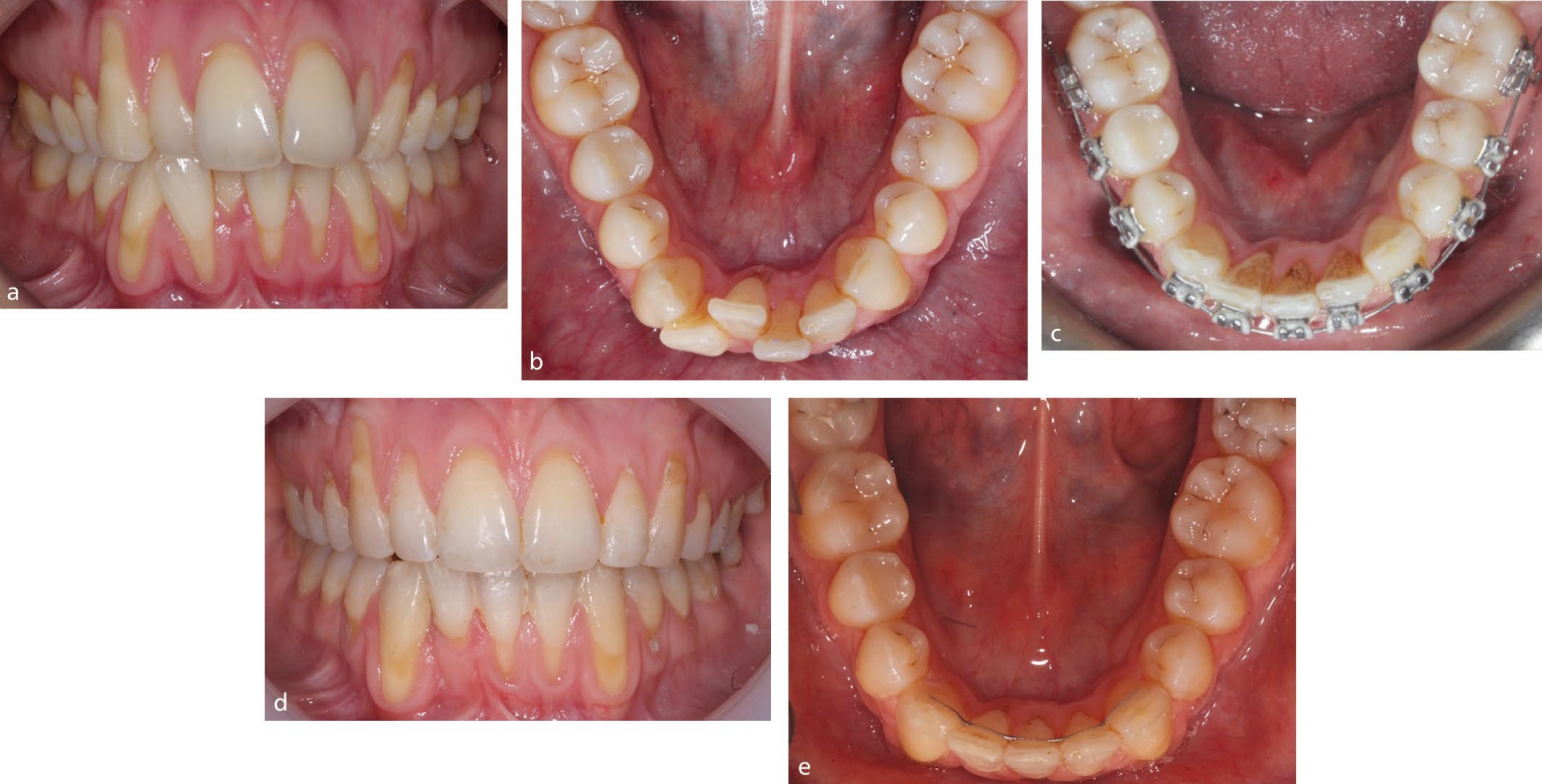
a, b) A 35-year-old woman presented with dual-arch crowding with thin periodontal phenotype and moderate lower anterior crowding. c) The lower right lateral incisor was labially excluded with significant associated recession. d, e) A decision was made to extract this tooth in order to avoid arch lengthening with a limited-objective plan to align both arches accepting a slight residual overjet

**Fig. 6:**
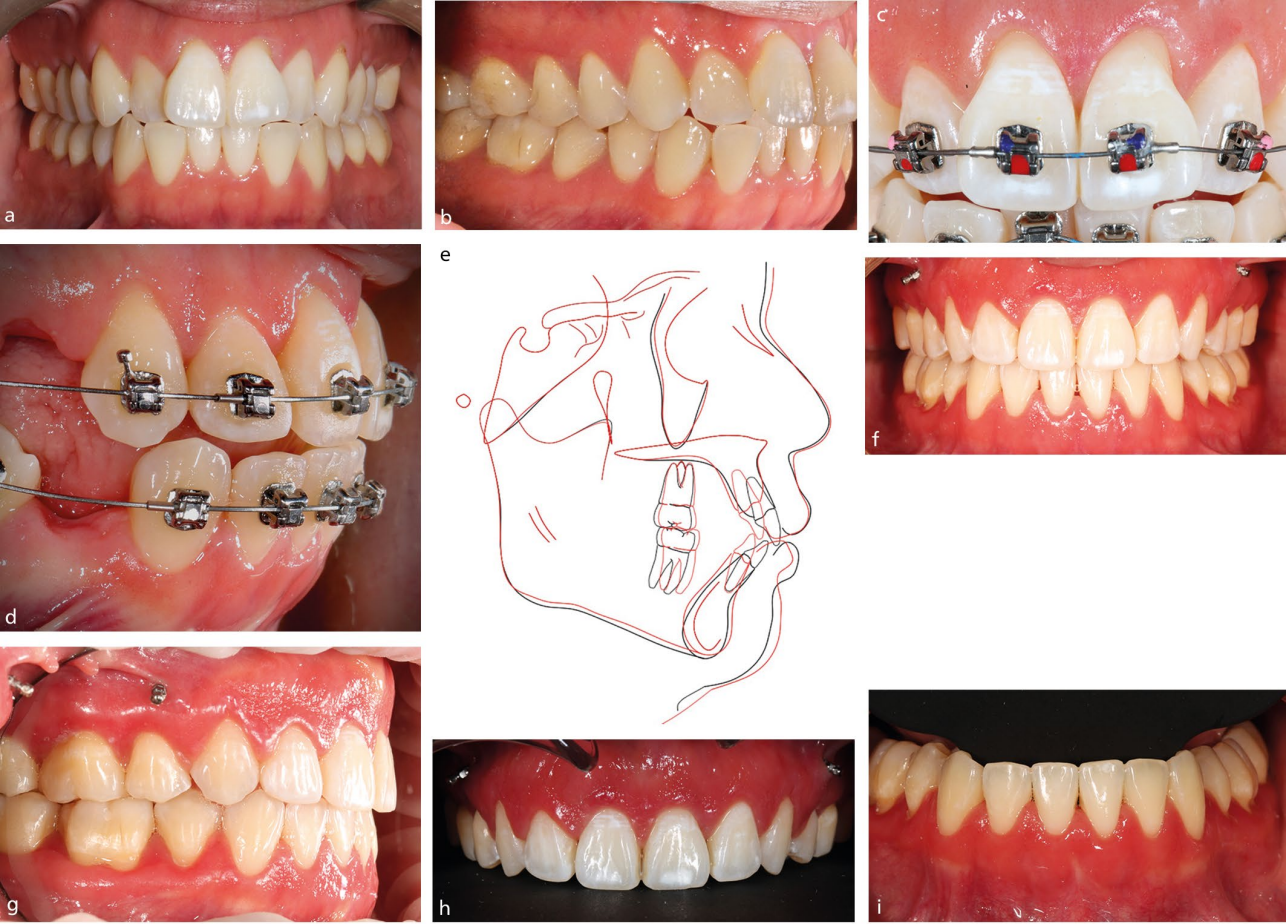
a, b) Bimaxillary proclination with associated crowding a patient with thin periodontal phenotype. c, d, e) Treatment was undertaken on an extraction basis with maximal anchorage in order to address the crowding and protrusion while retracing the dentition into the alveolar housing. f, g, h, i) A favourable gingival response to treatment was observed

As in the case of Stage III and Stage IV periodontal disease, the correct timing of space creation is an imperative. In particular, uncontrolled arch lengthening due to insufficient space may lead to displacement of the roots from the alveolar housing, further compromising gingival aesthetics and periodontal health (Fig. 5). Conversely, appropriate space creation and anchorage management may be harnessed to enhance gingival support by retracting the dentition into enhanced regions of bony support (Fig. 6). Formal quantification of space requirements is therefore advisable in order both to mitigate either under- or over-estimation of space needs.^27^ The latter may also be problematic due to the risk of excessive treatment duration and indeed failure to close extraction spaces. The likelihood of adjunctive use of inter-proximal reduction to enhance gingival aesthetics should also be accounted during extraction decisions.

### Local torque delivery

**Fig. 7:**
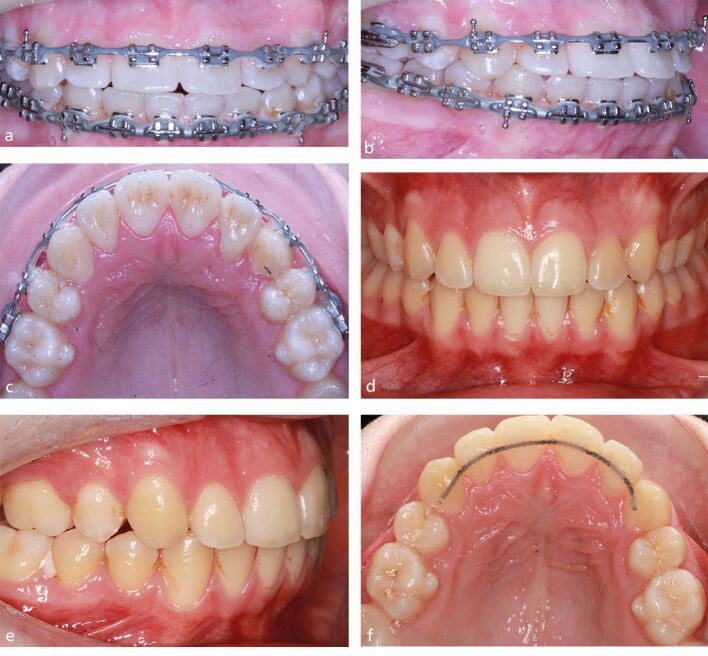
A 15-year-old male patient had been undergoing extraction-based orthodontic treatment in another practice. a, b, c) Significant flaring of the maxillary incisors had occurred with loss of torque and impingement of the apices on the lingual cortex. d, e, f) The existing fixed appliances were replaced with inversion of the maxillary incisal attachments introducing labial root torque, control of mesial angulation in the maxillary canines and sparing space creation being used to upright the incisors within cancellous bone over a period of eight months

**Fig. 8:**
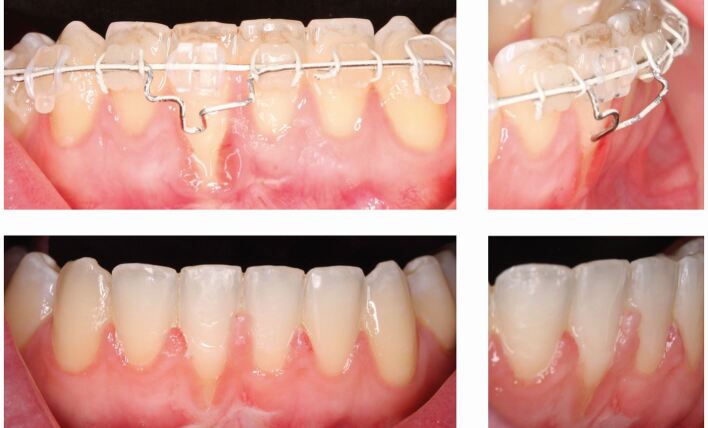
A transfer case presented with significant labial recession on lower right central incisor. An inverted mandibular premolar attachment was placed on the tooth to promote lingual root torque with an auxiliary wire formed in 0.018-inch stainless-steel added to augment this. Gingival grafting is planned following orthodontic treatment

Local torque delivery can be particularly important in the presence of thin periodontal phenotype promoting repositioning of the roots within the confines of cancellous bone. This can be achieved with fixed appliances or aligners with local bracket variations, wire bending and the use of auxiliaries. In view of the anatomical factors, local variations are typically required in the anterior region. Specifically, the torque prescription on the mandibular incisors can be reversed; for example, by inversion of MBT incisor attachments introducing (up to six degrees of) lingual root torque. Similarly, torque variations can be used in the maxillary incisor region to address torque issues and related periodontal consequences (Fig. 7). These local variations exert local effects following the engagement of rectangular archwires. Increasing the rigidity and dimensions of rectangular wires increases their potency with recognised geometric limitations associated with torque expression in a horizontal bracket slot. These shortcomings are compounded by wire and bracket flexibility, ligation mode, oversized brackets and undersized slots.^28^ On that basis, a number of solutions not reliant on the bracket-archwire interface exist including wire-bending (Fig. 8), proprietary adjuncts (eg Goodman springs) and use of other auxiliaries e.g., strategic positioning of elastomeric chain occlusal to the brackets to introduce retroclination.

### Gingival response

**Fig. 9:**
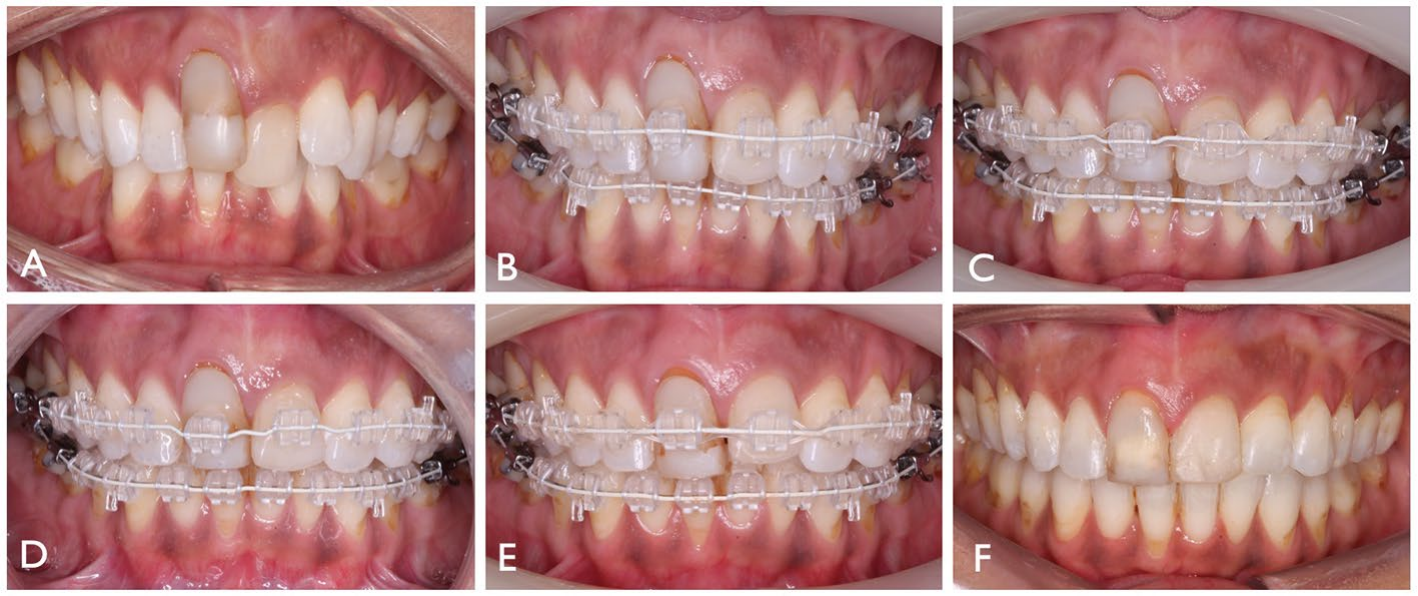
a) A 32-year-old woman presented having had a history of trauma to the maxillary right central incisor in adolescence. It had been assumed that this tooth was ankylosed with a marked gingival asymmetry. b, c, d, e) Based on a clinical assessment, ankylosis was excluded and fixed appliances were placed to gradually extrude 11 with periodic repositioning of the attachment, local extrusion bends and discing of the composite restoration to maintain aesthetics and permit unimpeded extrusion. f) The tooth was extruded over a 12-month period with placement of an interim maxillary fixed retainer before definitive restorative work

Orthodontic treatment may help to harmonise gingival levels with local recession, in particular, potentially impairing gingival aesthetics. Local vertical correction of tooth position can contribute to levelling of gingival margins, although faithful movement of gingival tissues cannot be guaranteed (Fig. 9). It is suggested that more gradual orthodontic movement favours commensurate movement of the gingival apparatus, although stretching of gingival tissues in the presence of thinner periodontal phenotype may be particularly uncertain. Notwithstanding this, marked improvements can be expected in most instances with gingival tissues moving approximately 80% relative to the tooth.^29^

### Old extraction sites

Old extraction sites, particularly in the presence of thin periodontal phenotype, may present a significant impediment to tooth movement. In particular, hour-glass shaped deformity with little cancellous bone inter-posed between the cortical plates are likely to be most resistant. When coupled with thin periodontal phenotype and pre-existing recession, a decision may be made to accept space locally or indeed to facilitate prosthetic replacement. Alternatively, space closure can be attempted with orthodontics alone or indeed with adjunctive periodontal treatments including bone grafting to promote space closure.^30^^,^^31^ Nevertheless, it is important to set realistic expectations in this respect being responsive to lack of associated progress.

## Conclusions

Orthodontic treatment in the presence of thin periodontal phenotype presents a diagnostic and clinical challenge. Specifically, combined orthodontic-periodontal input may be helpful in planning with strategic aims devised in order to safeguard periodontal health while producing acceptable orthodontic outcomes. Periodontal surgical procedures may be indicated before orthodontics particularly in recession defects with minimal (<1 mm) width of attached gingiva and gingival thickness. With careful planning, however, orthodontic treatment may offer a valuable adjunct to periodontal treatments in complementing orthodontic correction improving ‘pink aesthetics' while also reducing the risk of further periodontal breakdown.
